# CBAP promotes thymocyte negative selection by facilitating T-cell receptor proximal signaling

**DOI:** 10.1038/cddis.2014.474

**Published:** 2014-11-13

**Authors:** K-C Ho, Y-J Chiang, A C-Y Lai, N-S Liao, Y-J Chang, H-F Yang-Yen, J J-Y Yen

**Affiliations:** 1 Institute of Molecular Medicine, College of Medicine, National Taiwan University, Taipei, Taiwan; 2 Institute of Biomedical Sciences, Academia Sinica, Taipei, Taiwan; 3 Institute of Molecular Biology, Academia Sinica, Taipei, Taiwan

## Abstract

T-cell receptor (TCR)-transduced signaling is critical to thymocyte development at the CD4/CD8 double-positive stage, but the molecules involved in this process are not yet fully characterized. We previously demonstrated that GM-CSF/IL-3/IL-5 receptor common *β*-chain-associated protein (CBAP) modulates ZAP70-mediated T-cell migration and adhesion. On the basis of the high expression of CBAP during thymocyte development, we investigated the function of CBAP in thymocyte development using a CBAP knockout mouse. CBAP-deficient mice showed normal early thymocyte development and positive selection. In contrast, several negative selection models (including TCR transgene, superantigen staphylococcal enterotoxin B, and anti-CD3 antibody treatment) revealed an attenuation of TCR-induced thymocyte deletion in CBAP knockout mice. This phenotype correlated with a reduced accumulation of BIM upon TCR crosslinking in CBAP-deficient thymocytes. Loss of CBAP led to reduced TCR-induced phosphorylation of proteins involved in both proximal and distal signaling events, including ZAP70, LAT, PLC*γ*1, and JNK1/2. Moreover, TCR-induced association of LAT signalosome components was reduced in CBAP-deficient thymocytes. Our data demonstrate that CBAP is a novel component in the TCR signaling pathway and modulates thymocyte apoptosis during negative selection.

T-cell development—including cell differentiation, maturation, and selection—takes place in the thymus.^1^ During these processes, thymocytes receive various survival and/or death signals. For example, binding of stem cell factor^2^ and interleukin-7,^3,4^ and formation of the pre-T-cell receptor (TCR)^5^ and chemokine receptor CXCR4^6^ are critical for the survival and differentiation of the distinct subpopulation of CD4/CD8 double-negative (DN) thymocytes. In CD4/CD8 double-positive (DP) thymocytes, however, TCR signaling determines survival or death.^7^ Moderate signaling triggered by the interaction of TCR and self-peptide/major histocompatibility complex (MHC) supports DP thymocyte survival, a process called positive selection. On the other hand, DP thymocytes die when there is little or no TCR signaling, called death by neglect. Positively selected thymocytes commit to either the CD8 or CD4 single-positive (CD8SP or CD4SP) lineage. To prevent peripheral tissues from being attacked by autoreactive T lymphocytes, developing thymocytes undergo negative selection, a process that kills thymocytes exhibiting strong TCR reactivity toward self-antigens. At the end of the developmental process, SP thymocytes enter the circulation and become naïve T cells.^8^

Establishing tolerance to self is essential for shaping a well-functioning immune system, and thymic negative selection is a crucial step in this process.^9^ After TCR activation by the self-peptide/MHC complex, SRC protein kinases are activated, which recruit ZAP70. Two critical substrates of ZAP70, LAT and SLP76, are then activated to organize formation of the proximal signalosome, which is responsible for transducing a series of kinase cascades that in turn lead to the release of secondary messengers and activation of RAS and mitogen-activated protein kinase (MAPK) pathways.^10^ In response to strong TCR signals, MAPKs are suggested to trigger death pathways mediated by the BCL-2 family member BIM and the orphan nuclear receptor NUR77, respectively.^9, 11, 12, 13^ Both BIM and NUR77 act on BCL-2,^14,15^ which likely results in mitochondrial dysfunction followed by thymocyte death.^9^ Optimal formation and signaling of the LAT signalosome during negative selection promotes efficient elimination of thymocytes that can potentially attack the organism itself, thereby preventing the development of autoimmunity.

We previously reported that GM-CSF/IL-3/IL-5 receptor common *β*-chain-associated protein (CBAP) plays a dual role in *in vitro* cell apoptosis^16^ and *in vivo* T-cell migration and adhesion.^17^ In T cells, CBAP is a component of the *β*1-integrin complex and is involved in ZAP70-mediated VAV1 phosphorylation. The interaction of CBAP and VAV1 increases following chemokine stimulation, whereas the interaction of CBAP and ZAP70 is chemokine-independent.^17^ The CBAP-ZAP70 interaction implies a potential involvement of CBAP in TCR signaling, which prompted us to study the potential function of CBAP in T-cell development. We found that CBAP transcripts are highly expressed in thymocyte populations during various stages of T-cell lineage development. We investigated the role of CBAP in thymocyte development and the molecular mechanism by which CBAP modulates TCR signaling. Our data suggest that CBAP is a novel component of the LAT signalosome and plays a positive role in thymocyte negative selection.

## Results

### Normal early thymocyte development and positive selection in CBAP-deficient mice

To further characterize the function of CBAP in T-cell development, we measured CBAP mRNA levels in thymocytes and peripheral T cells and found that CBAP expression is much higher in thymocytes than in peripheral T cells. The amount of CBAP transcript was ~10-fold lower in naïve and activated T cells than in DP thymocytes, but was several-fold higher in DN, CD8SP, and CD4SP thymocytes than in DP thymocytes (Supplementary Figure S1a).

We then investigated the function of CBAP in thymocyte development using a gene knockout (KO) strategy. Mice lacking CBAP had normal thymus appearance and size (data not shown). The total number of thymocytes was not significantly different between wild-type (WT; ~1.72 × 10^8^) and CBAP-deficient (~1.45 × 10^8^) mice (Supplementary Figure S1b). The percentage of TCR*β*^high^ thymocytes was also comparable in CBAP-deficient and WT mice (Supplementary Figure S1c), indicating no obvious defect in TCR rearrangement and maturation. Moreover, we found no difference in the percentage of DN, DP, CD4SP, and CD8SP thymocyte populations (Supplementary Figure S1d) or in the percentages of DN1 to DN4 subpopulations (DN1, CD25^−^CD44^+^; DN2, CD25^+^CD44^+^; DN3, CD25^+^CD44^−^; DN4, CD25^−^CD44^−^; Supplementary Figure S1e). Notably, the relative expression of CBAP mRNA was higher in DN (~3.2-fold), CD8SP (~5.0-fold), and CD4SP (~7.6-fold) thymocytes than in DP thymocytes (Supplementary Figure S1a). This expression pattern drove us to further investigate whether CBAP loss affects any developmental checkpoints.

**Figure 1:**
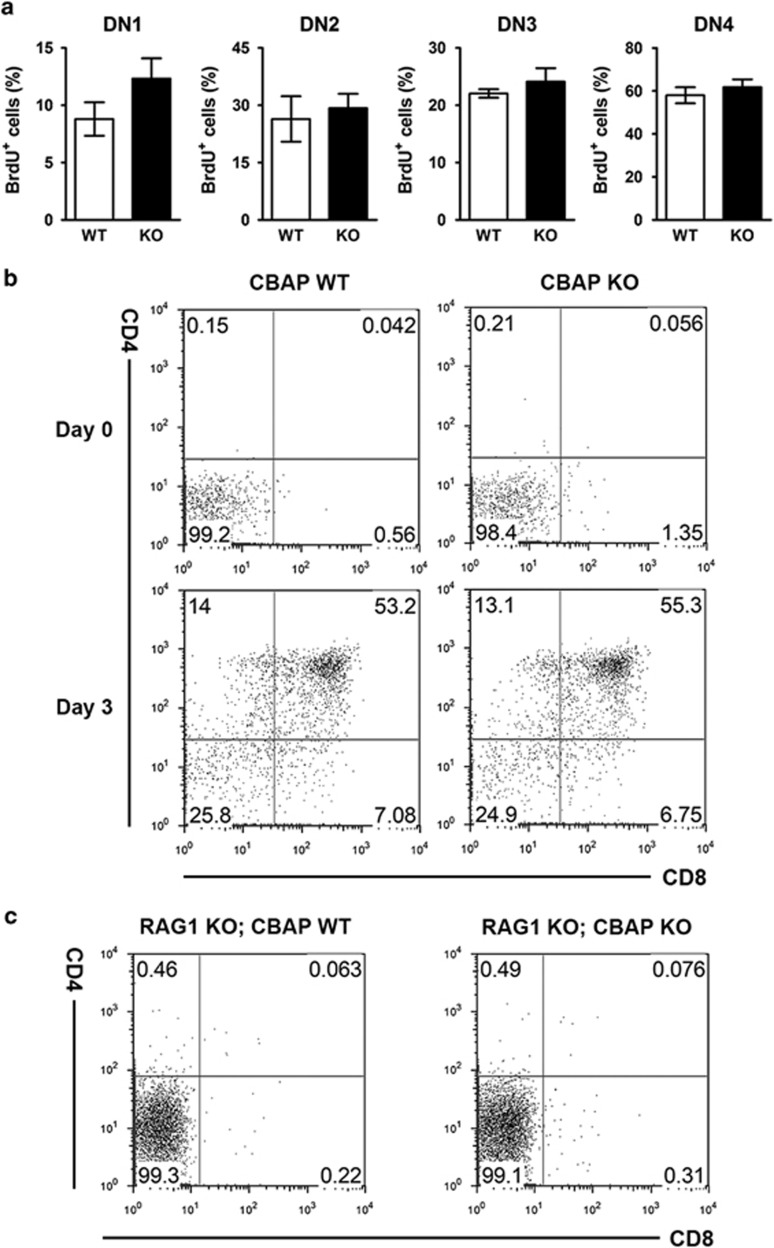
Normal early thymocyte development in CBAP-deficient mice. (**a**) Percent of proliferating cells among DN1-4 thymocytes. Four-week-old littermates were injected with 1 mg 5-bromo-2′-deoxyuridine and killed 2 h post injection. Total isolated thymocytes were stained for lineage markers (B220, CD3, CD4, CD8, CD11b, Gr-1, NK1.1, TER119) plus CD25 and CD44. Lineage-negative thymocytes (negative for all lineage markers) were gated out and further divided into four subpopulations, DN1 to DN4. The percentage of cells with incorporated 5-bromo-2′-deoxyuridine was analyzed within each lineage-negative DN subpopulation and plotted as mean±S.D. (*n*=4). (**b**) Flow cytometric analysis of thymocytes subjected to DN-to-DP *in vitro* differentiation culture. DN thymocytes were isolated from 4-week-old littermates and co-cultured with OP9-DL4 cells in the presence of 1 ng/ml FLT3-ligand and 1 ng/ml IL-7. At day 3, thymocytes were collected, stained for CD8 and CD4, and analyzed by flow cytometry (*n*=3). (**c**) Thymocyte development in RAG1-deficient mice. Thymocytes from 4-week-old RAG1/CBAP double-deficient mice and RAG1-deficient littermates were stained for CD8 and CD4 and analyzed by flow cytometry (*n*=3)

At the DN stage, massive proliferation normally occurs prior to *β*-selection. Thus, we assessed the possible alteration of proliferation in DN subpopulations by measuring 5-bromo-2′-deoxyuridine incorporation. No significant differences were detected between WT and CBAP-deficient mice in any subpopulation (Figure 1a). We also examined the *in vitro* differentiation capability of DN thymocytes when co-cultured with OP9-DL4 cells.^18^ There was no significant difference between WT and CBAP-deficient thymocytes in this *in vitro* assay system (Figure 1b). Because CBAP has a pro-apoptotic function in a variety of cell lines,^16^ we next examined whether it is involved in apoptosis of DN thymocytes that fail to rearrange the TCR*β* gene, a process that is at least partly FADD-mediated.^19^ We generated RAG1 and CBAP double-deficient mice and found that CBAP deficiency could not rescue cells from death caused by a failure of TCR*β* rearrangement.^19^ The CD8/4 profiles of these double-deficient mice were similar to those of RAG1-deficient mice (Figure 1c), indicating that CBAP is dispensable for thymocyte death at this checkpoint. Taken together, these results demonstrate that CBAP is not required in early thymocyte development.

**Figure 2:**
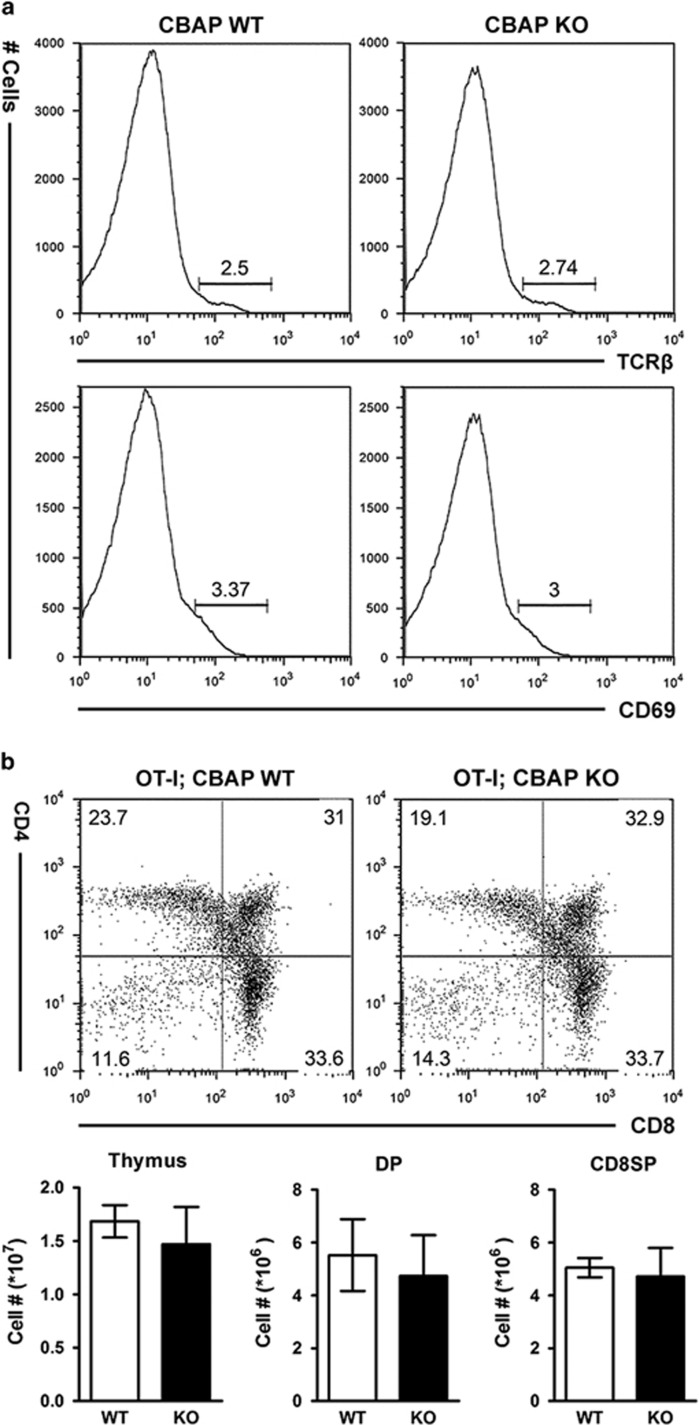

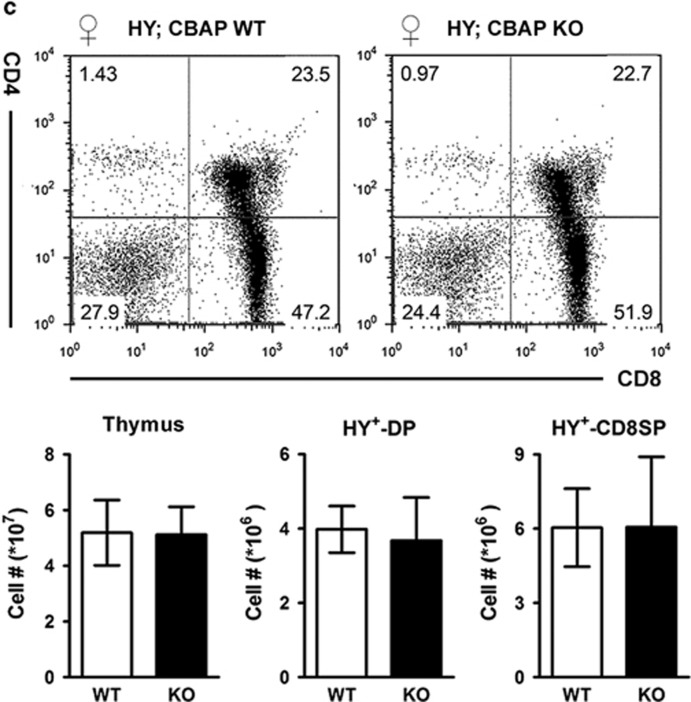
Normal positive selection in CBAP-deficient thymocytes. (**a**) Total thymocytes from 4-week-old littermates were stained for CD8, CD4, TCR*β*, and CD69. Histograms of TCR*β* and CD69 on gated DP thymocytes are shown (*n*=4). (**b**,**c**) Surface staining of CD8 and CD4 on thymocytes from 4-week-old littermate OT-I (**b**) and female HY (**c**) mice. Upper panels: Flow cytometric profiles. Lower panels: Absolute numbers of total, DP, and CD8SP thymocytes of mice carrying each TCR transgene were calculated as total cell number times the percentage of each thymocyte subpopulation (*n*=5−7)

To investigate whether CBAP is required for positive selection, we analyzed expression of the maturation markers TCR*β* and CD69 in DP thymocytes. Expression of these markers was not significantly different between WT and CBAP-deficient mice, indicating that the proportion of DP thymocytes undergoing positive selection is comparable (Figure 2a). We used TCR transgenic mice to examine the functional role of CBAP in positive selection. The percentage of CD8SP thymocytes in OT-I and female HY TCR transgenic mice was significantly increased (~33% in OT-I, ~50% in HY TCR females) compared with non-transgenic mice (~2%, Supplementary Figure S1d).^20, 21^ When OT-I and female HY TCR transgenic mice were crossed with CBAP-deficient mice, their offspring had comparable CD8/CD4 expression profiles (Figures 2b and c). These data suggest that CBAP is not essential for positive selection.

### Attenuated negative selection in CBAP-deficient mice

**Figure 3:**
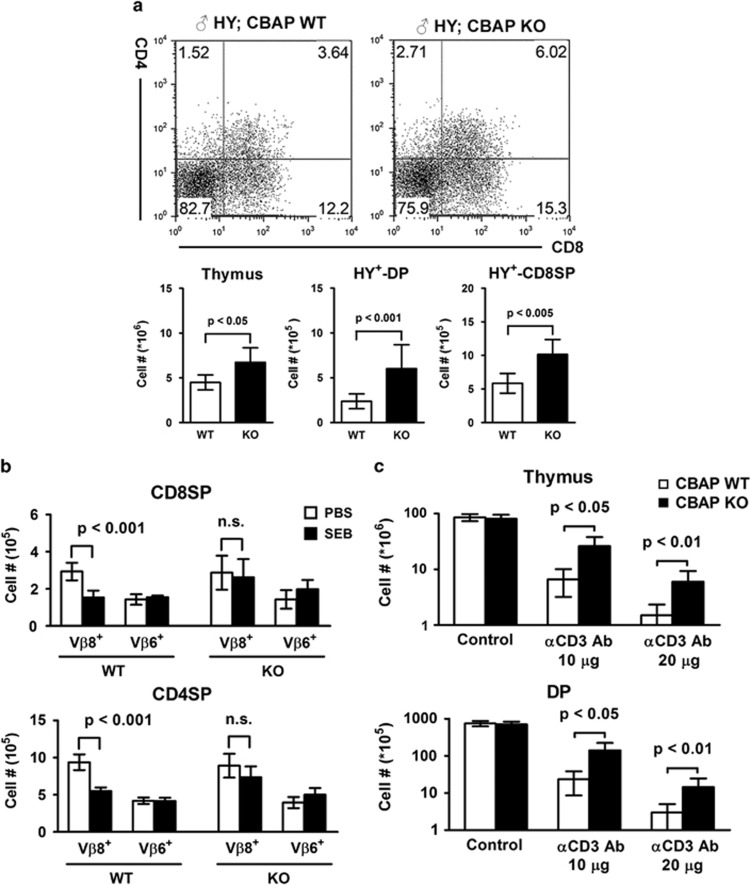
Compromised negative selection in CBAP-deficient thymocytes. (**a**) Thymocytes from 4-week-old male HY littermates were stained for CD8, CD4, HY TCR, and PI. Upper panel: Flow cytometric profiles. Lower panel: Absolute numbers of total, DP, and CD8SP thymocytes from mice carrying the HY TCR transgene (*n*=7) were calculated as in Figures 2b and c. (**b**) Seven-week-old littermates were injected with 10 *μ*g SEB or PBS (vehicle) on day 0, 2, and 4. Mice were killed on day 6. Isolated thymocytes were counted and stained for CD8, CD4, V*β*8, and V*β*6, and analyzed by flow cytometry. Numbers of V*β*8^+^ and V*β*6^+^ thymocytes in CD8SP (upper) and CD4SP (lower) populations were plotted as mean±S.D. (*n*=7). n.s., not significant. (**c**) Eight-week-old littermates were injected with 10 *μ*g (*n*=4) or 20 *μ*g (*n*=5) anti-CD3 Ab or PBS (*n*=5). Mice were killed after 40 h. Isolated thymocytes were counted, stained for CD8, CD4, and PI, and analyzed by flow cytometry. Numbers of total and DP thymocytes were plotted as mean±S.D.

We examined the role of CBAP in negative selection using the HY TCR transgenic mouse model. As reported previously, in male mice, HY TCR^+^ thymocytes are nearly absent because of the presence of endogenous male-specific HY autoantigens.^22^ Compared with the control group, male CBAP KO; HY TCR mice harbored elevated numbers of total (~1.5-fold), HY^+^-DP (~2.5-fold), and HY^+^-CD8SP (~1.7-fold) thymocytes (Figure 3a), indicating that CBAP is required for the efficient deletion of autoreactive thymocytes in HY TCR transgenic mice.

To further confirm the role of CBAP in negative selection, we injected WT and CBAP-deficient mice with superantigen staphylococcal enterotoxin B (SEB). As previously reported,^23^ this treatment induced the selective deletion of V*β*8^+^, but not V*β*6^+^, thymocytes in SP populations (Figure 3b). Whereas SEB treatment led to a 50% elimination of V*β*8^+^ thymocytes in the WT CD8SP population, CBAP-deficient mice showed resistance to SEB treatment, with no significant reduction (*P*=0.63) in V*β*8^+^ thymocytes. Similar protective effects of CBAP deficiency on SEB-induced V*β*8^+^ thymocyte deletion were observed in the CD4SP population (Figure 3b). The number of V*β*6^+^ thymocytes was not decreased in either population after SEB treatment (Figure 3b).

Finally, we examined TCR crosslinking-induced thymocyte deletion by injecting mice with anti-CD3 antibody (Ab). This *in vivo* treatment triggered strong TCR signals and led to thymocyte elimination (Figure 3c), as shown previously.^15^ After injection of anti-CD3 Ab, the numbers of total and DP thymocytes in both WT and CBAP-deficient mice were reduced in a dose-dependent manner. However, the number of total and DP thymocytes that remained was significantly higher in CBAP-deficient mice than in WT mice (~4- and ~5-fold, respectively; Figure 3c). Although anti-CD3 Ab and superantigen treatment cannot completely recapitulate the condition of TCR activation under natural conditions, and expression of the HY TCR transgene is earlier than its physiological expression time point because of the promoter used, loss of CBAP attenuated thymocyte deletion consistently in all three models examined.

### Abatement of TCR-induced BIM expression and JNK phosphorylation in CBAP-deficient thymocytes

**Figure 4:**
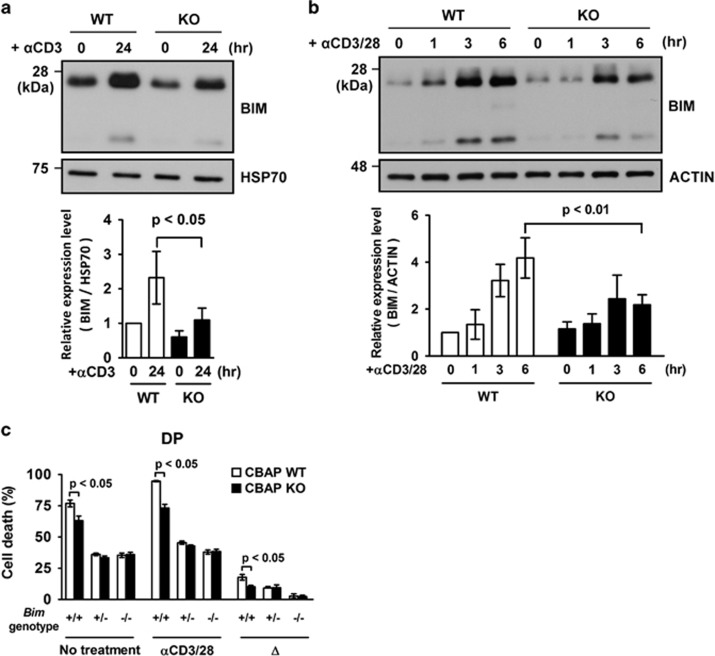
Reduced TCR-induced BIM accumulation in CBAP-deficient thymocytes. (**a**) Eight-week-old littermates were injected with 20 *μ*g anti-CD3 Ab. Total thymocytes were harvested without treatment or 24 h post injection. BIM and heat shock protein 70 (HSP70) were detected in cell lysates by western blotting. BIM expression in each group was quantified relative to HSP70 and plotted as mean±S.D. (*n*=4). (**b**) Thymocytes were cultured on plates coated with anti-CD3 (10 *μ*g/ml) and anti-CD28 (20 *μ*g/ml) Abs. After the indicated times, cells were collected, and BIM and actin were detected by western blotting. Expression of BIM was quantified relative to actin (*n*=4). (**c**) Thymocytes from indicated genotypes were cultured onto plates coated with anti-CD3 (1 *μ*g/ml) and anti-CD28 (5 *μ*g/ml) Abs or uncoated plates. After 24 h, thymocytes were collected and stained for CD8, CD4, annexin V, and PI. Survival of DP thymocytes was assessed by flow cytometry with statistical analysis. Specific TCR-induced apoptosis (▵) was calculated as the percentage of dead DP thymocytes in test cultures minus that in untreated cultures. Results were plotted as mean±S.D. (*n*=4)

To explore the molecular pathways by which CBAP participates in negative selection, we first examined whether the loss of CBAP alters the induction of two known death effectors, BIM and NUR77, after TCR crosslinking.^9^ We examined cell lysates from remaining thymi of WT and CBAP KO mice 24 h after Ab injection *in vivo,* and showed that there was a significant upregulation of BIM protein expression in WT cells, and that KO cells had a significantly lower level of BIM than WT cells (Figure 4a, upper panel, 24 h post injection). TCR crosslinking using anti-CD3/28 Abs in *in vitro* cultured thymocytes from WT mice induced not only profound cell death (see below), but also profound elevation of BIM expression (Figure 4b), which recapitulated the phenotype of *in vivo* anti-CD3 Ab injection (Figures 3c and 4a). Once again, TCR crosslinking of KO thymocytes *in vitro* led to significantly reduced BIM induction compared with crosslinking in WT thymocytes (Figure 4b, upper panel, 3 or 6 h post treatment). These results were highly reproducible, and quantification analysis of *in vivo* and *in vitro* TCR crosslinking assays by densitometry showed that loss of CBAP led to a 53% (Figure 4a, lower panel) or 48% (Figure 4b, lower panel) reduction of BIM accumulation, respectively. In addition, as shown in Supplementary Figure S2, TCR crosslinking using anti-CD3/CD28 Abs caused the drastic increase of *nur77* mRNA expression in WT cells, as previously shown.^24^ Loss of CBAP had no effect on the expression of *nur77* mRNA upon TCR crosslinking (Supplementary Figure S2). Therefore, we focused further investigation on the function of CBAP in BIM induction.

To investigate whether BIM expression is involved in CBAP-modulated thymocyte death, we generated BIM/CBAP double-deficient mice. We first compared the effect of the *BIM* genotype on the extent of *in vitro* thymocyte cell death using culture medium alone. In the *CBAP* WT background, loss of one or two copies of *BIM* had a similarly protective effect (*BIM*^+/+^, 77% *BIM*^+/−^, 36% *BIM*^−/−^, 35% Figure 4c, no treatment), suggesting that the cytokine-free induced death signal is very sensitive to BIM protein level. Loss of CBAP showed only modest protection in *BIM*^+/+^ thymocytes (reduced from 77 to 63%, *P*<0.05), and no protection in *BIM*^+/−^ (33%) or *BIM*^−/−^ (36%) thymocytes (Figure 4c, no treatment), indicating that the survival benefit of CBAP deficiency can be covered by the loss of one copy of *BIM*. When TCR was activated, the death of control *BIM*^+/+^ thymocytes was elevated to 95% after 24 h of anti-CD3/28 Ab crosslinking in cytokine-free culture medium. Loss of *BIM* showed a dose-dependent protective effect (*BIM*^+/−^, 46% *BIM*^−/−^, 38% Figure 4c, *α*CD3/28), especially when crosslinking-induced thymocyte death (Δ) was calculated, which was 18% for *BIM*^+/+^, 9.2% for *BIM*^+/−^, and 2.8% for *BIM*^−/−^ thymocytes, and was consistent with a previous report.^15^ CBAP deficiency provided significant protection from TCR-crosslinking-induced death in *BIM*^+/+^ thymocytes, where it was reduced from 95 to 73% (Figure 4c, *α*CD3/28). This protective effect was also still significant when crosslinking-induced thymocyte death was calculated (10% death for *BIM*^+/+^). However, deletion of CBAP did not show further protection when one or two copies of *BIM* was deleted (9.4% death for *BIM*^+/−^; 2.4% for *BIM*^−/−^; Figure 4c, Δ), suggesting that the BIM-mediated death pathway is the predominant pathway CBAP is involved.

**Figure 5:**
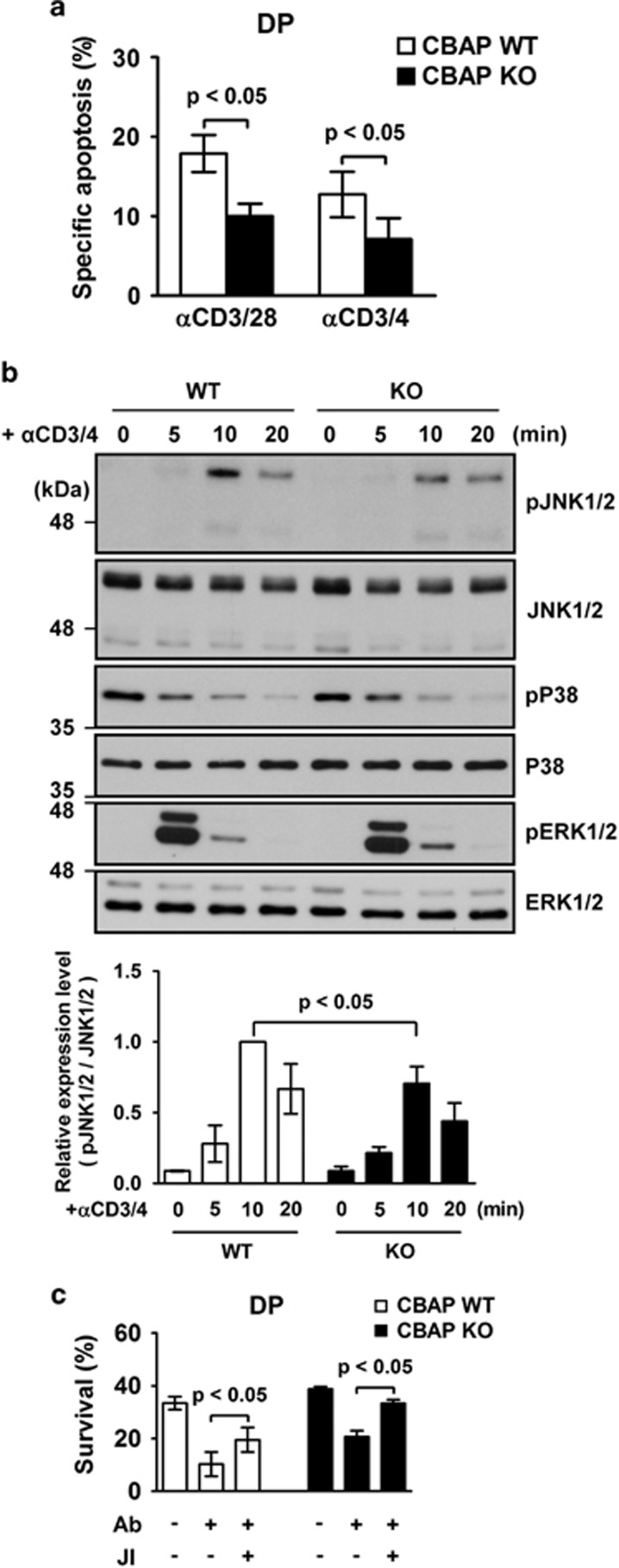
Reduced JNK phosphorylation in CBAP-deficient thymocytes upon TCR crosslinking. (**a**) Thymocytes were cultured on plates coated with the indicated Abs. After 24 h, specific TCR-induced apoptosis was analyzed by flow cytometry and calculated as in Figure 4c (*n*=4). (**b**) Thymocytes were stimulated by anti-CD3 (5 *μ*g/ml) and anti-CD4 (5 *μ*g/ml) Abs for the indicated times. Lysates were prepared and levels of unphosphorylated and phosphorylated JNK1/2, P38, and ERK1/2 proteins were analyzed by western blotting (upper panel). Quantitative results for pJNK1/2 expression are shown in the lower panel (*n*=3). (**c**) Thymocytes with or without JNK inhibitor SP600125 pretreatment (JI, 5 *μ*M, 30 min before Ab crosslinking) were cultured and analyzed as in Figure 4c. Survival of DP thymocytes was plotted as mean±S.D. (*n*=4)

To further refine the role of CBAP in this signaling pathway, we compared the effect of anti-CD3/4 Abs crosslinking in the *in vitro* TCR crosslinking assay, and again found that the CBAP deficiency significantly protected thymocytes from death by CD3/28 or CD3/4 crosslinking (Figure 5a). This suggests that CBAP is mainly involved in the signaling pathway initiated by TCR/CD3 complex and co-receptor molecule CD4, and less likely through the co-stimulatory molecule CD28.

We next explored activation of the MAPKs JNK1/2 and P38, which are implicated in BIM upregulation following *in vitro* anti-CD3/4 crosslinking.^13^ In CBAP-deficient thymocytes, phosphorylation of JNK1/2 was decreased by ~29% at 10 min after crosslinking (Figure 5b, upper and lower panels), whereas phosphorylation of P38 and ERK1/2 was not affected (Figure 5b, upper panel). The observation that levels of ERK1/2, which has been implicated in signaling for thymocyte positive selection,^25^ were not affected by absence of CBAP is consistent with our findings in TCR transgenic mouse models that CBAP is not required for positive selection (Figures 2b and c). We then used the JNK inhibitor SP600125 to block TCR-induced thymocyte death,^26^ and found that survival of thymocytes during TCR crosslinking was increased in both the presence and absence of CBAP (Figure 5c, PI low and annexin V low sections), indicating that CBAP acts upstream of JNK. These data suggest that the loss of CBAP attenuates optimal JNK activation and BIM induction following TCR crosslinking, and supports *in vivo* observations that loss of CBAP leads to a reduction in thymocyte negative selection after strong TCR triggering (Figure 3).

### Involvement of CBAP in TCR proximal signaling

**Figure 6:**
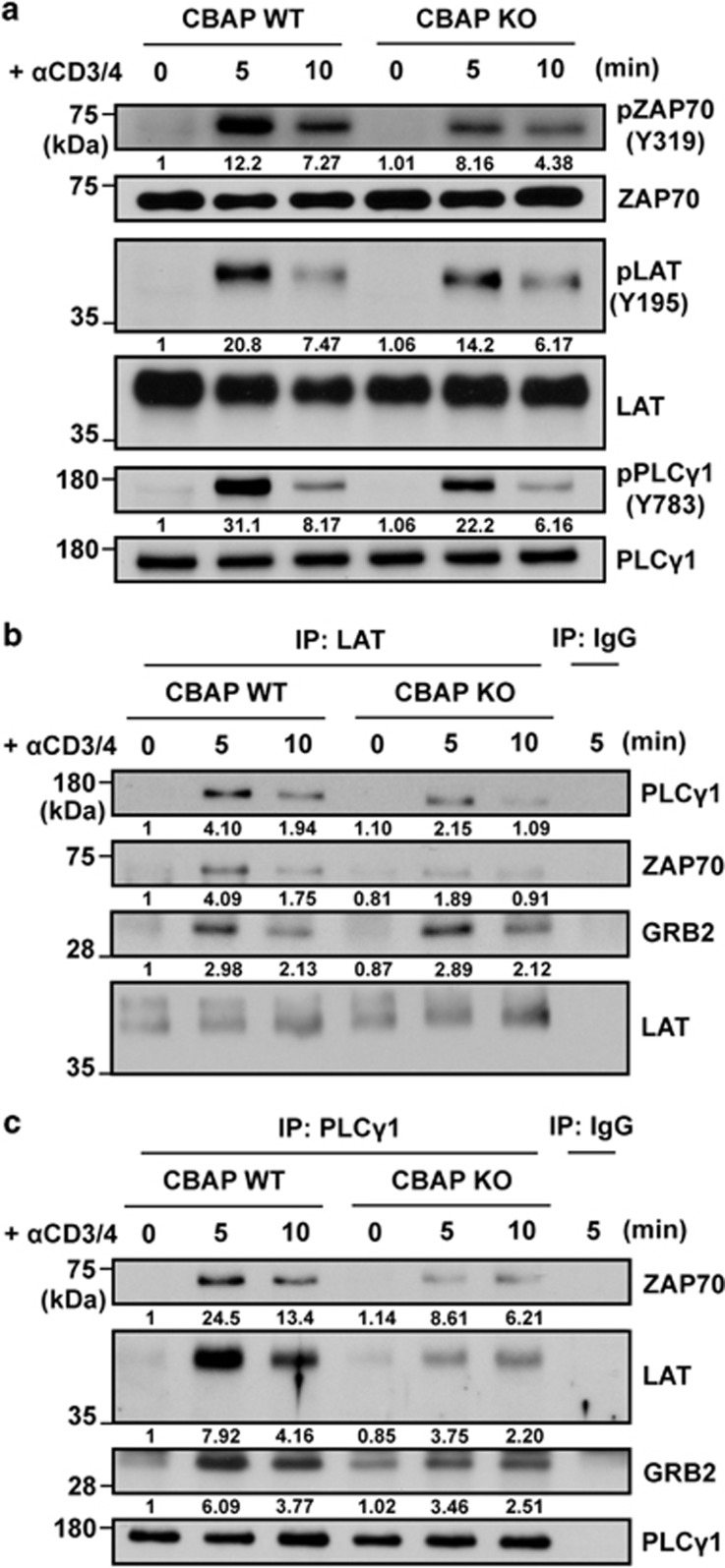
Involvement of CBAP in TCR-induced signaling complex formation. Thymocytes were stimulated with anti-CD3 (5 *μ*g/ml) and anti-CD4 (5 *μ*g/ml) Abs for the indicated times. (**a**) Proteins were prepared from cell lysates, and unphosphorylated and phosphorylated proteins were detected by western blotting (*n*=3). Phosphorylation sites are indicated in parentheses. (**b**,**c**) Immunoprecipitation was performed using anti-LAT (**b**) or anti-PLCγ1 (**c**) Ab with IgG controls, and western blots were probed with Abs for PLC*γ*1, ZAP70, LAT, and GRB2 (*n*=4)

Finally, we investigated how CBAP modulates BIM induction and JNK phosphorylation after TCR crosslinking. On the basis of our previous observation that CBAP associates with ZAP70 in Jurkat T cells,^17^ we speculated that CBAP may be a novel component involved in TCR proximal signaling. Using co-immunoprecipitation in Jurkat T cells, we detected ZAP70, LAT, and GRB2, but not SLP76 or PLC*γ*1, in CBAP-containing complexes (data not shown). Therefore, we examined phosphorylation of TCR proximal components in CBAP-deficient thymocytes, where JNK phosphorylation was diminished upon *in vitro* TCR crosslinking. TCR-induced phosphorylation of ZAP70, LAT, and PLC*γ*1 was decreased by ~30% at 5 min after crosslinking in CBAP-deficient thymocytes (Figure 6a). We then used anti-LAT and anti-PLC*γ*1 Abs for co-immunoprecipitation experiments to investigate the integrity of the LAT signalosome in thymocytes following TCR crosslinking. In CBAP-deficient thymocytes, LAT-ZAP70 and LAT-PLC*γ*1 associations were reduced by ~50% at 5 min after TCR crosslinking, whereas LAT-GRB2 associations remained unaffected (Figure 6b). Loss of CBAP also led to attenuated PLC*γ*1-ZAP70, PLC*γ*1-LAT, and PLC*γ*1-GRB2 associations upon TCR crosslinking (Figure 6c). These data suggest that CBAP is a previously unidentified integral component of the LAT signalosome and is required for transducing optimal TCR signaling.

## Discussion

In the present study, we characterized the pro-apoptotic role of CBAP in thymocyte development. Our findings are consistent with the *in vitro* pro-apoptotic function of CBAP we reported previously.^16^ We performed several experiments to demonstrate the influence of CBAP in thymocyte deletion. First, CBAP deficiency led to increased survival of residual autoreactive thymocytes in male HY TCR transgenic mice (Figure 3a). Second, CBAP was important for effective deletion of V*β*8^+^ thymocytes after superantigen SEB treatment (Figure 3b). Third, the loss of CBAP reduced the efficiency of thymocyte deletion after anti-CD3 Ab injection (Figure 3c). Further analysis showed that JNK1/2 activation and BIM induction were compromised in CBAP-deficient thymocytes after TCR crosslinking (Figures 4 and 5). These data suggest that negative selection stimuli operate through a pathway that requires CBAP for optimal thymocyte deletion.

There are limitations to each of the three models used here to assess the role of CBAP in natural negative selection. First, although anti-CD3 Ab treatment *in vivo* is a fast way to induce thymocyte deletion, it cannot fully mimic natural TCR triggers, and may induce DP thymocyte death via cytokines or hormones produced by activated T cells. To exclude such nonspecific death, we performed *in vitro* TCR crosslinking using plate-bound anti-CD3/CD28 Abs and showed that the effect of CBAP on thymocyte death is cell autonomous (Figure 5a). Second, superantigen treatment is a model used to demonstrate deletion of specific thymocyte subsets, but it suffers from the fact that TCR is not activated by a physiological MHC/peptide complex. Third, although the HY TCR transgenic mouse model is a good system to evaluate thymocyte elimination by a natural antigen, expression of the HY TCR transgene in T-cell development occurs earlier than normal because of the promoter used, and the percentage of responders is higher than normal, which may yield non-physiological outcomes. Despite these limitations, the loss of CBAP consistently mitigated thymocyte deletion in all three models supports a role of CBAP in natural negative selection.

TCR signaling is critical for the death or survival of developing thymocytes. In addition to the previously reported association between CBAP and ZAP70 in resting Jurkat T cells,^17^ the defects in thymocyte deletion observed here support the potential involvement of CBAP in TCR signaling. Strong TCR-MHC/peptide interactions are known to activate MAPKs, which are suggested to trigger BIM- and NUR77-mediated death effector pathways.^9^ JNK is reported to regulate pro-apoptotic functions of BIM,^27, 28^ and is implicated in promoting thymocyte death through the induction of BIM expression.^9, 12^ Upon TCR crosslinking, CBAP-deficient thymocytes manifested less JNK activation and BIM induction than WT thymocytes (Figures 4 and 5). Previous studies demonstrated that BIM plays a key pro-apoptotic role in the DP-to-SP transition, including negative selection and death by neglect.^15, 29, 30^ Similarly, our results demonstrate that loss of CBAP decreased the efficiency of thymocyte apoptosis via negative selection (Figure 3) and death by neglect (Figure 4c, no treatment and *α*CD3/28), which may result from reduced BIM accumulation following TCR crosslinking (Figures 4a and b). Furthermore, in the BIM KO background, additional CBAP deficiency did not further increase thymocyte survival after TCR crosslinking, strongly suggesting that CBAP modulates TCR-induced cell death via a BIM-dependent pathway. On the other hand, the loss of CBAP did not significantly alter the abundance or activation of ERK1/2, which is critical for positive selection,^25^ consistent with the normal phenotype in CBAP-deficient mice during this developmental process (Figure 2).

It is known that the integrity of TCR signaling is essential for the fate of developing thymocytes. Mice harboring deficiencies of key TCR signaling components (e.g., LCK,^31^ ZAP70^32^, and LAT^33^) have developmentally blocked phenotypes. On the other hand, recent studies have reported that deficiencies of THEMIS,^34, 35, 36^ TESPA1^37^, and ZFAT^38^ impair positive and/or negative selection because of partial attenuation of TCR signaling. The mild defects in CBAP-deficient mice suggest an accessory role for CBAP in assembly of the TCR-induced LAT signalosome (Figure 6). Among the associations we examined, CBAP deficiency led to reduced LAT-ZAP70, LAT-PLC*γ*1, and PLC*γ*1-ZAP70 association, but did not affect LAT-GRB2 association. In the deduced amino acid sequence of CBAP contains an SH2-like domain (^−2^T/S-x-x-x-x-V/I^+3^;^39^), but no kinase/catalytic domain has been annotated. Considering that extensive phosphorylation is involved in TCR proximal signaling, CBAP may associate with these components in a phosphorylation-dependent manner and serve as an adaptor or scaffold protein to facilitate formation of the LAT signalosome by modulating protein-protein interactions. Although the mechanisms by which CBAP selectively modulates TCR-induced JNK phosphorylation are not yet understood, it is remarkable that CBAP is required for the discrimination of differential TCR stimuli to transduce corresponding downstream signaling.

Recent studies have suggested that actin rearrangement during TCR signaling is required to achieve complete thymocyte selection.^40^ Besides guiding thymocytes to the proper thymic location for selection,^41, 42^ migration/adhesion events could cooperate with TCR signaling in thymocyte development. Although the mechanisms by which chemokine signaling might influence negative selection are not fully understood, several studies have demonstrated cross-talk between chemokines and TCR signaling.^43, 44^ For instance, mice lacking chemokine CCR7 manifest impaired thymocyte negative selection.^45, 46^ Consistent with our previous study, we have found that CBAP-deficient SP thymocytes have attenuated chemotaxis toward CCL21 in a transwell assay (unpublished data). In addition, we have observed aberrant distribution of SP thymocytes in the medullary region of the CBAP-deficient thymus (unpublished data). Thus, in addition to its direct involvement in TCR signaling, CBAP may modulate negative selection indirectly by facilitating the interaction of thymocytes and medullary thymic epithelial cells.

Recently, a series of studies demonstrated an unexpected observation that deficiency in TCR signaling components may manifest immunodeficiency that accompanies autoimmunity, inflammatory diseases, and/or increased IgE production.^47^ Here, we report defective thymic negative selection in CBAP-deficient mice via impairment of the assembly of the LAT signalosome and disruption of TCR signaling (Figure 6). Intriguingly, hyporeactivity of CBAP-deficient peripheral T cells has also been detected in a DNFB-induced contact hypersensitivity model (unpublished data), suggesting that the modulatory role of CBAP in TCR signaling may be required for balance between immunogenic and tolerogenic TCR functions. Therefore, the potential involvement of CBAP in complex immune cross-talk and human clinical presentation warrant further characterization of CBAP-deficient mice in various disease models.

## Materials and Methods

### Mice

CBAP-deficient mice were described previously.^17^ Mice harboring the BIM deficiency and OT-I and HY TCR transgenes were purchased from Jackson Laboratory (Bar Harbor, ME, USA). All mice had a C57BL/6 background and were housed in the Taiwan Mouse Clinic. All mouse experiments were performed in accordance with guidelines approved by the Institutional Animal Care and Utilization Committee of Academia Sinica.

### Flow cytometry

Freshly isolated or cultured thymocytes were separated into single-cell suspensions and stained for various surface markers. Abs (eBioscience, San Diego, CA, USA) used for staining were conjugated to FITC, PE, PE-Cy7, or APC and raised against: CD4 (GK1.5), CD8*α* (53-6.7), TCR*β* (H57-597), CD69 (H1.2F3), B220 (RA3-6B2), CD3*ɛ* (145-2C11), TCR-V*α*2 (B20.1), TCR-V*β*5 (MR9-4), TCR-V*β*6 (RR4-7), TCR-V*β*8 (KJ16), NK1.1 (PK136), TER119 (TER-119), Gr-1 (RB6-8C5), CD11b (M1/70), HY TCR (T3.70), CD25 (PC61.5), and CD44 (IM7). For cell proliferation and cell death experiments, the 5-bromo-2′-deoxyuridine flow kit (BD Biosciences, San Jose, CA, USA) and annexin V apoptosis kit (BioVision, Milpitas, CA, USA) were used to detect 5-bromo-2′-deoxyuridine incorporation and dead cells, respectively. Data were collected on a FACSCanto analyzer (BD Biosciences) and analyzed using FlowJo software (TreeStar, Ashland, OR, USA).

### Quantitative RT-PCR

Analysis was performed on RNA from cultured thymocytes or thymocytes freshly isolated using a fluorescence-activated cell sorter or magnet-activated cell sorter (Miltenyi Biotec, Bergisch Gladbach, Germany). RNA was extracted using an RNeasy Mini kit (Qiagen, Venlo, The Netherlands), and cDNA was prepared from 2 *μ*g RNA using SuperScript III reverse transcriptase (Invitrogen, Carlsbad, CA, USA). Quantitative RT-PCR was performed using Power SYBR GREEN PCR Master Mix (Invitrogen) and the 7500 Real Time PCR System (Invitrogen) under standard conditions. Primer sets for detecting mRNA levels of *nur77*^48^ and *gapdh*^49^ were described previously. Relative fold expression was calculated by the 2^−ΔΔ*C*^_T_ method.^50^

### *In vitro* differentiation assay

Thymocytes were co-cultured with OP9-DL4 cells in the presence of 1 ng/ml FLT3-ligand (PeproTech, London, UK) and 1 ng/ml IL-7 (PeproTech) as previously described.^18^ After 3 days, cells were stained for CD4, CD8, and PI, and analyzed by flow cytometry.

### *In vivo* SEB treatment

The procedure for *in vivo* SEB treatment was modified from the one previously described.^23^ Briefly, mice were injected intraperitoneally with 10 *μ*g SEB (Sigma-Aldrich, St. Louis, MO, USA) or PBS on days 0, 2, and 4. On day 6, the absolute number of thymocytes was determined by hemocytometry. Thymocytes were stained for CD4, CD8, V*β*6, V*β*8, and PI, and analyzed by flow cytometry.

### TCR crosslinking assay

The procedure for TCR crosslinking was described elsewhere.^15^ For *in vivo* experiments, mice were injected intraperitoneally with 10 or 20 *μ*g anti-CD3 Ab (145-2C11) or PBS. After 40 h, total thymocytes were counted and analyzed by flow cytometry. For *in vitro* assays, thymocytes were cultured on anti-CD3 and anti-CD28 (37.51) Ab-coated plates, and each Ab was used at the indicated concentration. To measure thymocyte apoptosis, cells were collected and analyzed by flow cytometry after 24 h of culture. To determine expression levels of BIM and *nur77*, cells were lysed for extraction of total protein and RNA at the indicated times. To analyze TCR signaling in thymocytes, the stimulation procedure was modified slightly from a previous protocol.^51^ Briefly, thymocytes were incubated with biotinylated anti-CD3 Ab (5 *μ*g/ml, 145-2C11) and anti-CD4 Ab (5 *μ*g/ml, GK1.5, BioLegend, San Diego, CA, USA) on ice for 15 min. Prewarmed streptavidin (10 *μ*g/ml, Sigma-Aldrich) was used to crosslink the Ab-labeled cells for various time periods at 37 °C. Cells were lysed for immunoprecipitation and/or protein detection as indicated.

### Immunoprecipitation and western blotting

Thymocytes were stimulated or left untreated as indicated in each experiment. Cells were collected and lysed in buffer containing 50 mM Tris-HCl (pH 7.4), 150 mM NaCl, 1 mM EGTA (pH 8), and 0.2% NP-40. Protease inhibitor cocktail (Sigma-Aldrich) and PhosSTOP (Roche Molecular Diagnostics, Pleasanton, CA, USA) were added to the lysis buffer according to the user's manual. For IP, Protein G Mag Sepharose Xtra (GE, Fairfield, CT, USA) was used. Immunoprecipitates were washed twice with lysis buffer, twice with 0.5 M LiCl in 50 mM Tris (pH 7.6) and twice with water, as published previously.^52^ Abs were obtained as follows: (p-)MAPK Family Ab Sampler Kit (Cell Signaling Technology, Danvers, MA, USA); ZAP70, GRB2, and pPLC*γ*1(Y783) (Abcam, Cambridge, UK); PLC*γ*1 (Abcam and Merck Millipore, Billerica, MA, USA); LAT, pLAT(Y191/Y195), and pZAP70(Y319) (Merck Millipore); BIM (ProSci, Poway, CA, USA); HSP70 (Santa Cruz Biotechnology, Dallas, TX, USA); and actin (Sigma-Aldrich).

### Quantification and statistical analysis

Statistical analysis was performed using a nonparametric two-tailed Mann-Whitney test and visualized using GraphPad Prism 5 software (GraphPad Software, La Jolla, CA, USA).

## Abbreviations

CBAP: GM-CSF/IL-3/IL-5 receptor common *β*-chain associated protein
DN: double-negative
DP: double-positive
SP: single-positive
WT: wild type
KO: knockout
TCR: T-cell receptor
MHC: major histocompatibility complex
MAPK: mitogen-activated protein kinase
Ab: antibody
SEB: staphylococcal enterotoxin B
