# Supplementary figures and images for: CBAP promotes thymocyte negative selection by facilitating T-cell receptor proximal signaling
Source: Cell Death Dis. 2014 Nov 13;5(11):e1518–. doi: 10.1038/cddis.2014.474 (PMC4260732; doi:10.1038/cddis.2014.474)

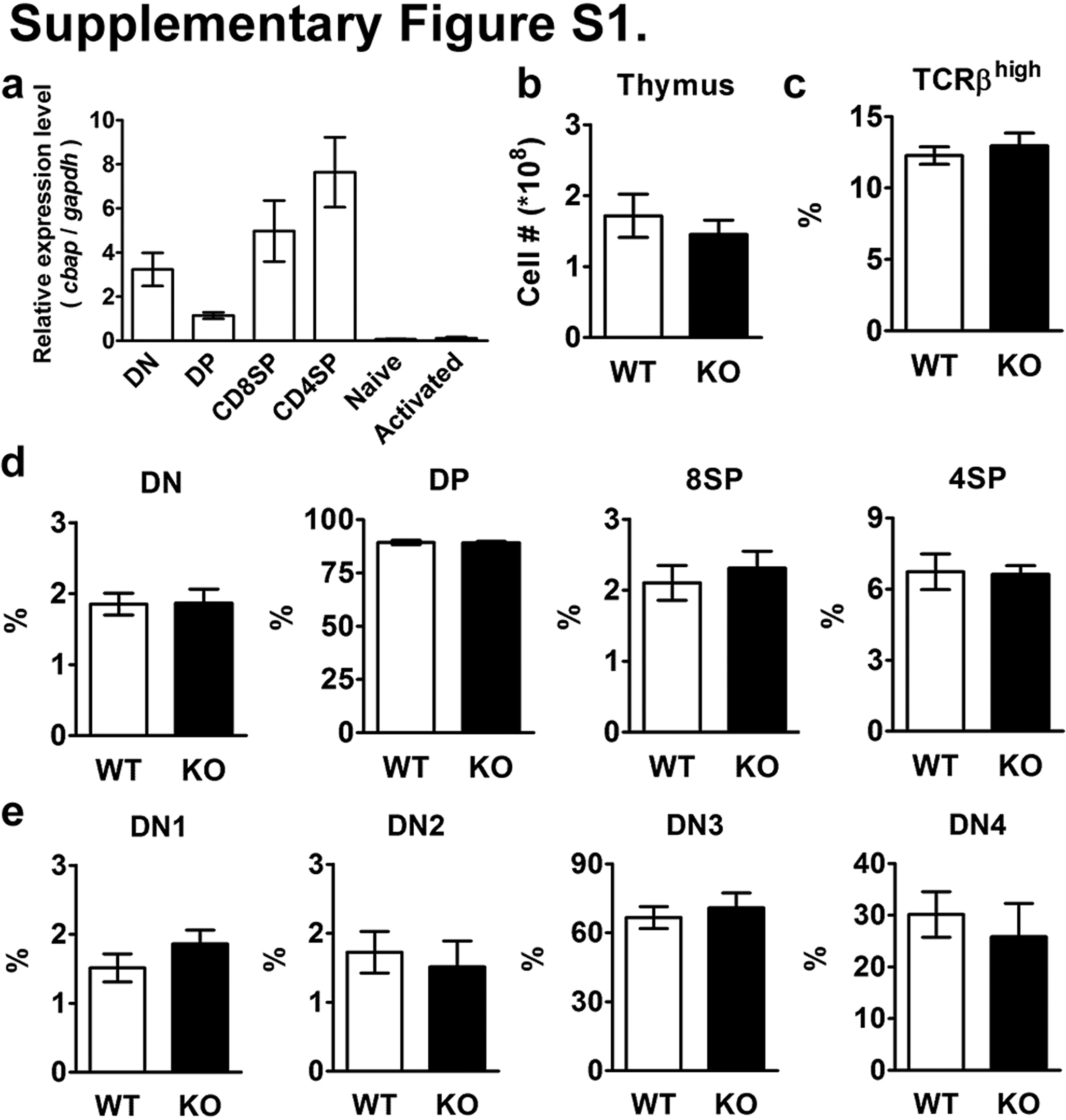

Supplement: Supplementary Figure S1 [file cddis2014474x1.tif]

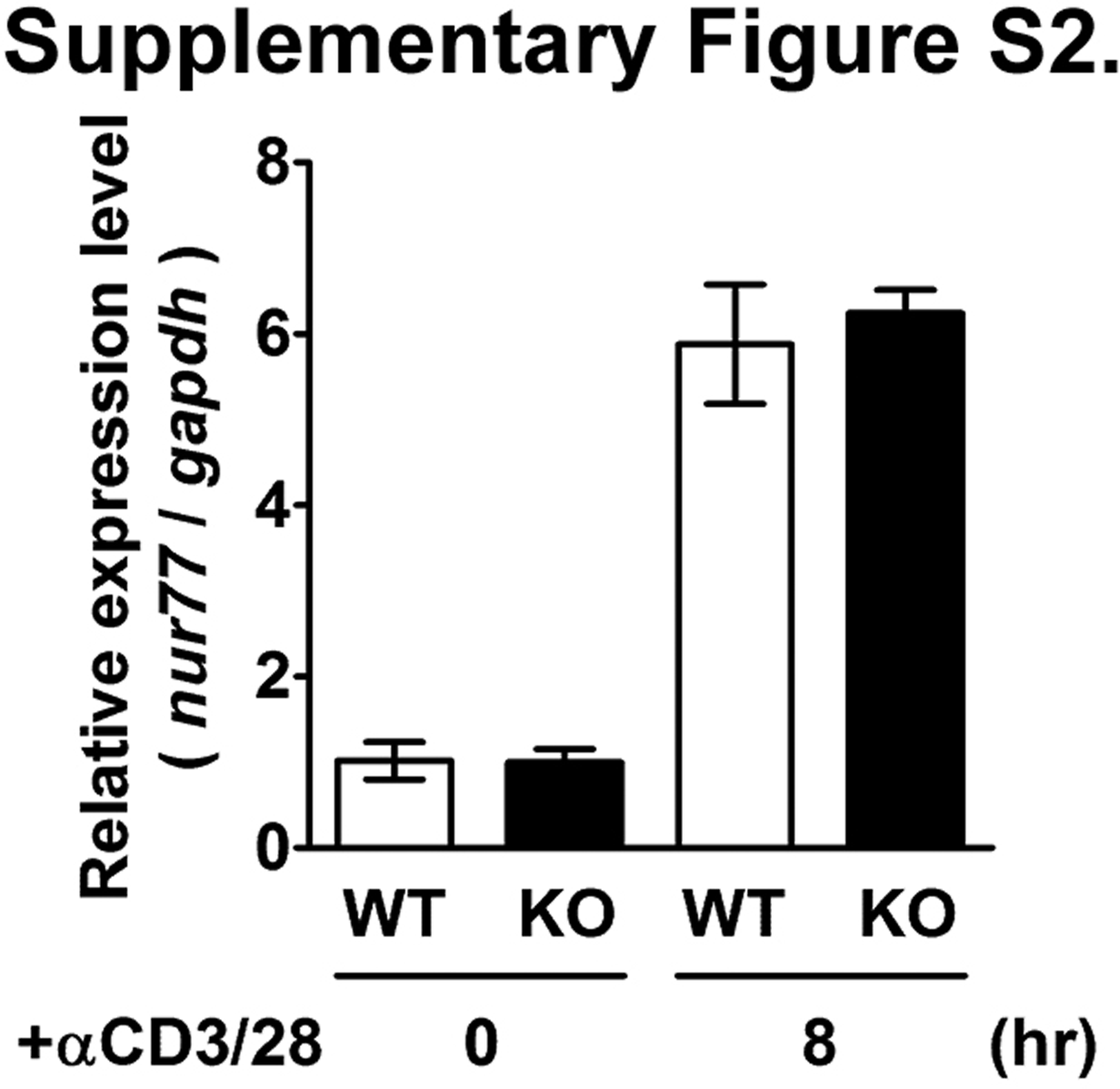

Supplement: Supplementary Figure S2 [file cddis2014474x2.tif]
