# Supplementary material for: CBAP promotes thymocyte negative selection by facilitating T-cell receptor proximal signaling
Source: Cell Death Dis. 2014 Nov 13;5(11):e1518–. doi: 10.1038/cddis.2014.474 (PMC4260732; doi:10.1038/cddis.2014.474)
Supplement: Supplementary Figure Legends [file cddis2014474x3.doc]

**Supplementary information**

**Supplementary Figure 1.** Characterization of thymocytes in CBAP-deficient mice. (**a**) Quantitative RT-PCR (Q-PCR) analysis of *cbap* mRNA expression in each population of T cell lineage. DN, DP, CD4SP, and CD8SP thymocytes were sorted by FACS, and peripheral naïve CD90.2^+^ T cells were purified by MACS. Some naïve T cells were further activated by plate-bound anti-CD3/28 Abs. Relative expression of *cbap* transcripts was detected by Q-PCR in mRNA samples purified from each cell population. *Gapdh* was used as an internal control (n = 4). (**b**) Total thymocytes from four-week-old littermates were counted by hemocytometry (n = 9). (**c-e**) Total thymocytes from four-week-old littermates were stained for TCRβ (c) or CD8 and CD4 (d). Lineage-negative (B220^–^ CD3^–^ CD4^–^ CD8^–^ CD11b^–^ Gr-1^–^ NK1.1^–^ TER119^–^) thymocytes were stained for CD25 and CD44 (e) and subdivided into DN1 (CD25^–^CD44^+^), DN2 (CD25^+^CD44^+^), DN3 (CD25^+^CD44^–^), and DN4 (CD25^–^CD44^–^) (n = 6). Results were plotted as mean ± SD.

**Supplementary Figure 2.** Induction of *nur77* mRNA expression upon *in vitro* TCR crosslinking. Thymocytes were treated as in Figure 4b. At the indicated times, thymocytes were harvested for detection of *nur77* and *gapdh* by quantitative RT-PCR (n = 4).
